# Agendas on Nursing in South Korea Media: Natural Language Processing and Network Analysis of News From 2005 to 2022

**DOI:** 10.2196/50518

**Published:** 2024-03-19

**Authors:** Daemin Park, Dasom Kim, Ah-hyun Park

**Affiliations:** 1 School of Media & Communication Sunmoon University Chungcheongnam-do Republic of Korea; 2 Home Visit Healthcare Team Expert Group on Health Promotion for Seoul Metropolitan Government Seoul Republic of Korea; 3 Tobacco Control Team Expert Group on Health Promotion for Seoul Metropolitan Government Seoul Republic of Korea

**Keywords:** nurses, news, South Korea, natural language processing, NLP, network analysis, politicization

## Abstract

**Background:**

In recent years, Korean society has increasingly recognized the importance of nurses in the context of population aging and infectious disease control. However, nurses still face difficulties with regard to policy activities that are aimed at improving the nursing workforce structure and working environment. Media coverage plays an important role in public awareness of a particular issue and can be an important strategy in policy activities.

**Objective:**

This study analyzed data from 18 years of news coverage on nursing-related issues. The focus of this study was to examine the drivers of the social, local, economic, and political agendas that were emphasized in the media by the analysis of main sources and their quotes. This analysis revealed which nursing media agendas were emphasized (eg, social aspects), neglected (eg, policy aspects), and negotiated.

**Methods:**

Descriptive analysis, natural language processing, and semantic network analysis were applied to analyze data collected from 2005 to 2022. BigKinds were used for the collection of data, automatic multi-categorization of news, named entity recognition of news sources, and extraction and topic modeling of quotes. The main news sources were identified by conducting a 1-mode network analysis with SNAnalyzer. The main agendas of nursing-related news coverage were examined through the qualitative analysis of major sources’ quotes by section. The common and individual interests of the top-ranked sources were analyzed through a 2-mode network analysis using UCINET.

**Results:**

In total, 128,339 articles from 54 media outlets on nursing-related issues were analyzed. Descriptive analysis showed that nursing-related news was mainly covered in social (99,868/128,339, 77.82%) and local (48,056/128,339, 48.56%) sections, whereas it was rarely covered in economic (9439/128,339, 7.35%) and political (7301/128,339, 5.69%) sections. Furthermore, 445 sources that had made the top 20 list at least once by year and section were analyzed. Other than “nurse,” the main sources for each section were “labor union,” “local resident,” “government,” and “Moon Jae-in.” “Nursing Bill” emerged as a common interest among nurses and doctors, although the topic did not garner considerable attention from the Ministry of Health and Welfare. Analyzing quotes showed that nurses were portrayed as heroes, laborers, survivors of abuse, and perpetrators. The economic section focused on employment of youth and women in nursing. In the political section, conflicts between nurses and doctors, which may have caused policy confusion, were highlighted. Policy formulation processes were not adequately reported. Media coverage of the enactment of nursing laws tended to relate to confrontations between political parties.

**Conclusions:**

The media plays a crucial role in highlighting various aspects of nursing practice. However, policy formulation processes to solve nursing issues were not adequately reported in South Korea. This study suggests that nurses should secure policy compliance by persuading the public to understand their professional perspectives.

## Introduction

### Controversy Over Nursing Legislation in South Korea

The COVID-19 pandemic has renewed the focus on the importance of nurses in South Korea’s health care system with a resultant increase in the interest in improving the nursing workforce structure, working conditions, and treatment [1]. Although the pandemic has fueled a movement to improve nursing policies in South Korea, nurses face difficulties in nursing-related policy activities because of various stakeholders and policy environments. As the nursing profession is regulated by the Medical Service Act enacted in 1951, rather than an independent law such as the Nursing Bill, there are concerns about the inadequacy of the legal status or rights of nurses and, consequently, their diminished role in the health care system.

In South Korea, the debate over nursing legislation has been ongoing for more than a decade. In 2005, Kim Sun-mi, a representative of the ruling Uri Party, first proposed nursing legislation—the Nursing Bill—in the Korean National Assembly; however, the bill was abandoned. In 2018, the bill was reintroduced and rejected and was eventually passed in the National Assembly in April 2023 as the Nursing Bill, shortly after the COVID-19 pandemic ended. However, President Yoon Suk Yeol vetoed the Nursing Bill, and the bill was rejected again in May 2023.

Compared with the early days of the COVID-19 pandemic, it is presently difficult to obtain a favorable public opinion among the public and other professions at the policy level. This may be because of the differences between the general public’s interests in nurses’ workforce structure, work, and treatment and the need for nursing-related policies [2,3].

### Research Context: The Importance of Media in Health Policy

Media coverage has an important impact on shaping public opinion on certain issues. The policy agenda–setting theory suggests that media coverage of an issue can influence the public agenda, which in turn can influence the policy agenda [4,5]. When the media continues to report on an issue, it draws public attention, which can lead to an increased public demand for action from policymakers. Thus, media coverage can be viewed as a form of valuable large-scale data that reflects the flow of social interest and changes in a particular phenomenon or issue. These data can be used to identify paradigm shifts in policies related to the issue.

With the aging population and increasing prevalence of chronic diseases, the medical burden would increase in South Korea. Furthermore, changes in the social environment and policies, such as the introduction of integrated nursing care services, affect the demand for nursing care. In this context, it is important for the public and policymakers to understand nursing care accurately. Media coverage has a significant impact on public perception of medical issues [6,7]. However, research on media coverage in nursing is still limited.

Previous studies focused on identifying the images of nurses in the media or analyzing individual issues [1,8-11]. Both the original Woodhull study conducted in 1997 and a subsequent study conducted in 2017 examined the representation of nurses in health-related news coverage. However, both studies had limitations in that the research involved analysis of news from a single year and focused solely on the health-related context without exploring nurses’ representation as news sources in all news sections [12,13].

Methodologically, several studies have used natural language processing (NLP) of news data, topic modeling, and network analysis [8-11,14]. However, most studies analyzed articles over a short period of less than a year [8-11,14]. This strategy has limited the scope of these studies and prevented them from capturing the entire range of issues faced by the nursing community. Additionally, the short timeframe of these studies makes it difficult to capture the long-term development and context of policymaking.

For collecting and NLP of data, we used BigKinds, the largest public news database in South Korea, comprising over 80 million news articles published starting in 1990 [15,16]. It was developed by the Korea Press Foundation, a governmental organization, to support the copyright business of news media. BigKinds is widely used for academic research of news analysis in various fields including nursing studies [9,11]. This study also conducted network analysis and qualitative analysis of quotes.

This study examined how nursing-related issues have changed over time by analyzing, by year and section, approximately 130,000 articles that were published in 54 major Korean media outlets over an 18-year period.

This study focuses on the sources of news articles and their quotes. Sources are key informants whom a reporter has heard directly from and therefore cited in the news for a direct quote [17]. In communication studies, sources are the most important elements of news writing. News is a description of what a source has said [18]. Thus, citing sources is a core factual practice in journalism, along with presenting figures and being present at the scene for direct observation [19].

This study aimed to examine the drivers of the social, local, economic, and political agendas emphasized in the media by the analysis of main sources and their quotes. This analysis revealed which nursing media agendas were emphasized (eg, social aspects), neglected (eg, policy aspects), and negotiated. Furthermore, this study determined the way in which policy formulation processes were not adequately reported.

### Research Questions

The research questions (RQs) of this study are as follows:

- RQ1: How many articles were reported by year and section?
- RQ2: Who have been the main sources of media coverage of nurses since 2005, by year and section?
- RQ3: What concerns did the health care community, in particular, nurses, medical doctors, and the Ministry of Health and Welfare (MOHW), pay more attention to and what were their common concerns?
- RQ4: What are the key issues of nursing controversies in the press in quotes?

RQ1 was researched by descriptive analysis; RQ2 was researched by analyzing the news source network; was researched RQ3 by analyzing the news source-topic network; and RQ4 was researched by an analysis of quotes from key sources. This strategy will allow us to see who has led the agenda-setting of nurse-related issues and to identify the agenda that has been emphasized by each section in the media.

## Methods

### Study Design

Descriptive analysis, NLP, and semantic network analysis were applied to analyze data collected from 2005 to 2022 using BigKinds (Korea Press Foundation), SNAnalyzer, and UCINET. First, BigKinds were used for the collection of data, descriptive analysis, automatic multi-categorization of news, named entity recognition (NER) of news sources, and extraction of quotes from news articles. Second, the main news sources were identified by conducting a 1-mode network analysis with SNAnalyzer. Third, the main agendas of nursing-related news coverage were examined by conducting the qualitative analysis of major sources’ quotes by section. Fourth, the common and individual interests of the top-ranked sources were analyzed through a 2-mode network analysis using UCINET.

### Data Collection

The collection of news data with NLP was conducted using BigKinds. We used 1 search term “간호사,” which means “nurse” in Korean. It is rare that an article about nurses does not mention “nurses”; rather, there may be an article that contains the word “nurse” but indirectly deals with nurses. Articles wherein nurses were peripherally featured were not excluded as they could provide context for nurse coverage.

This study collected data from sources and quotes in nurse articles published in South Korea from January 1, 2005, to December 31, 2022. In 2005, an independent nursing law was proposed for the first time in South Korea by a member of the National Assembly.

The news articles were collected from 54 major domestic media outlets, including national, economic, local, broadcasting, and specialized newspapers, and provided by BigKinds.

### Natural Language Processing

BigKinds provides various advanced NLP functions, including automatic article classification, NER, quote extraction, mapping of sources and quotes through semantic analysis, and auto-tagging and ranking of topics in each quote through topic modeling.

The news analyzed was selected from 4 sections—social, local, economic, and political—which are highly related to the policy aspects of nurses and contain numerous articles. The sections are automatically classified by BigKinds. Automatic classification overcomes the differences in naming sections between years and media outlets. Each article can be categorized into a maximum of 3 different sections. For instance, a political article dealing with regional issues can be classified into both political and local sections simultaneously.

Source extraction is accomplished through NER for names, affiliations, and job titles. According to the manual, BigKinds’ NER uses an algorithm that combines structured support vector machine and Bidirectional Encoder Representations from Transformers (BERT) with an *F*_1_-score of 91.5%.

We extracted quotes from BigKinds and mapped sources to their quotes through semantic analysis. This study focused on major sources’ quotes.

### Cleansing

Data cleansing was done in 2 stages. In the first stage, we removed incorrect sources based on character count, part of speech, and so on. Furthermore, we removed anonymous sources and remaining institutional sources. We kept sources who were labeled as a profession or group, such as “nurses” or “hospitals,” and who were closely related to the research topics.

The second round of cleansing was conducted after ranking the sources according to the news source network analysis described later. Given the nature of semantic networks, incorrect results in NLP tend to be marginalized by low rankings. Therefore, rather than refining the entire data set from the beginning, it is more efficient to refine only the top-ranked sources who are candidates for the analysis.

In this study, we first performed a second round of cleansing on the top 100 sources. If there was a synonym for the name of an organization due to using abbreviations or anaphoras, we kept the higher-ranked source and unified them with the same name. Through this process, the top 20 news sources by section and year were selected. All the different sources that were tied around the 20th position of the cutoff were included. Finally, 445 sources that had made the top 20 list at least once by year and section were selected. We present the top 20 sources in each section based on cumulative yearly degree centrality, summarized briefly.

### Descriptive Analysis

BigKinds provides the number of articles related to searching keywords by year and section. The total number of articles is deduplicated but the sum of the number of articles by section can exceed the total number of articles because each article is categorized into up to 3 sections.

### Semantic Network Analysis

In this study, thousands of sources were cited in the articles related to nursing for decades. Thus, semantic network analysis was performed to rank sources by year and section and determine the relationship between sources and topics. Specifically, news source network analysis and news source-topic network analysis were conducted.

The news source network is an undirected 1-mode network with sources as nodes and article cooccurrences as edges. The importance of each source is evaluated based on the degree centrality of the news source network. A source with a high degree centrality appears in many articles and is discussed in many agendas with many different sources [16]. We also provide descriptive information (size, number of edges, and density) of networks.

A program called SNAnalyzer was used for network analysis [16,20]. SNAnalyzer analyzes multiple Excel files simultaneously with folder-to-folder input and output and provides data cleansing, file name standardization, degree centrality, tie strength, ranking, and descriptive statistics for up to 1,048,575 nodes per file.

NetDraw of UCINET software was used to visualize the news source-topic network. News source-topic network analysis is a 2-mode undirected network with sources and quote topics as nodes and quote cooccurrences as edges. The strength of networks shows the number of times the sources mentioned each topic. News source–topic network analysis can be used to determine which topics are of common interest to sources, and which are of interest only to specific sources. This study focused on 3 groups of sources: nurses, doctors, and the MOHW. Each group was merged according to their affiliations to compare interests at the organizational level.

### Quote Analysis

This study also performed quote analysis as a qualitative analysis to deeply understand the context of nursing in news media. In quote analysis, we focused on quotes uttered by the top 445 sources of each year and each section and contained the word “nursing” as a topic in the quotes. Because most top sources were highly cited, analyzing the quotes alone from the top sources resulted in analyzing a substantial number of citations. The number of quotes containing “간호 (nursing)” was 26,926. The total number of deduplicated quotes was 162,316.

### Ethical Considerations

Due to the nature of the research involving nonhuman participants, this study was exempt from review by the institutional review board of SM University (202302-001-1). Researchers collected publicly available existing data.

## Results

### Descriptive Analysis

The total number of articles was 128,339, excluding duplicate articles. The number of articles per section was 99,868 for the social section, 48,056 for the local section, 9439 for the economy section, and 7301 for the political section. A given article was classified as a duplicate in up to 3 sections.

**Figure 1:**
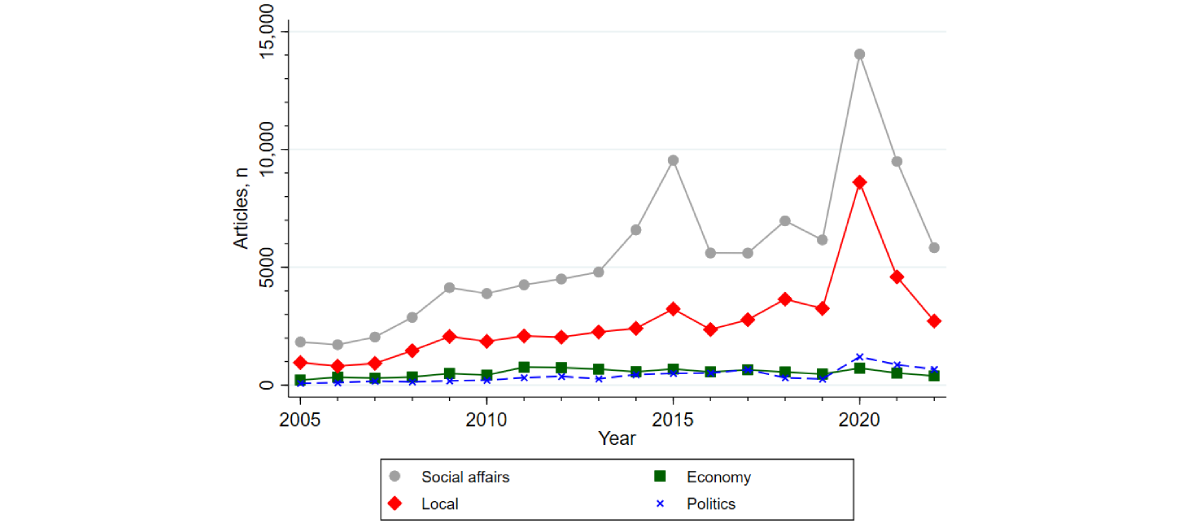
Number of articles by year and news section.

Figure 1 shows the frequency distribution of the articles by year and section. The years 2009, 2015, and 2020 saw surges in news articles corresponding to the swine flu, Middle East respiratory syndrome (MERS), and COVID-19 epidemics, respectively. In 2018, a “burning” incident—a metaphor for bullying in the workplace—occurred among nurses at the Asan Medical Center in Seoul. This incident led to a rapid increase in the number of articles that focused on the nursing culture. These results suggest that the media paid considerable attention to the response to new infectious diseases and accidents in the nursing profession.

### Semantic Network Analysis: News Source Network Analysis

#### Overview

There were 72 news source networks. We present the size, number of edges, and density of these networks as network-level descriptive statistics in Multimedia Appendix 1.

Multimedia Appendices 2-5 present the top 20 sources by centrality value, which represents the level of connectivity in terms of the years and sections. Each source’s rank is determined as the sum of the links. Sources related to the social aspect include incidents and accidents, medical and health issues, education, labor and welfare, trade unions, and activities in civil society. Local governments, such as the Seoul metropolitan government, police, and courts, serve as major sources for the social departments of media companies. Therefore, nurses and police are recognized as important sources in the social sector.

The main sources representing each section are briefly presented in the following sections with tables in the Multimedia Appendices. The interests of main sources are discussed in more detail in the sections on news source-topic network analysis and quote analysis.

#### Social Section

“Nurses (the total sum of the degree centrality is 2091)” were the most important sources in the social section of the media, especially in crucial cases such as the 2009 H1N1 influenza epidemic (degree centrality in 2009 is 65), the 2013 Jinju Medical Center incident (sum of degree=74), the 2015 MERS incident (sum of degree=117), and the 2016 nurse workplace bullying and medical malpractice controversy (sum of degree=130). The above-described importance persisted through 2018 (sum of degree=176) and was further emphasized by the COVID-19 pandemic, which began in 2020 (sum of degree=351) in a different context. During the COVID-19 pandemic, governmental sources, such as Central Disaster Management (242 in 2021), came to the forefront.

The period when the “hospital” source (the total sum of the degree centrality is 1790) was considered important coincided with the period when the “nurse” source served as an important source. This was mainly because the hospital was both a nurse’s workplace and a medical malpractice site. The importance of the “police” source significantly increased in 2018 (sum of degree=158) when the issue of nurse suicide by bullying and medical malpractice gained social prominence.

#### Local Section

“Hospital” (577 in total) is the most important source in the local section, especially in 2009 (sum of degree=42), 2014-2015 (sum of degree=34 and 38), and 2017-2021 (sum of degree=54, 36, 39, 103, and 70). During these periods, various impertinent issues were highlighted, including overwork, poor working conditions, and subsequent turnover within the hospital setting. Trade unions have emerged as crucial sources and have thereby contributed to the discourse surrounding the identified issues.

Related to the “labor union” (207 in total), specific incidents that garnered significant attention included the decision to close the Jinju Medical Center in South Gyeongsang Province in 2013 (sum of degree=37), which was criticized for the perceived moral hazard potential. Additionally, the general strike organized by the health care union in 2021 (sum of degree=36) in regard to the expansion of doctor numbers and the adjustment of the patient-to-nurse ratio became a topic of extensive discussion.

The media have played a vital role in disseminating information and shaping public opinion during the COVID-19 pandemic. Of particular interest were the decisions made by the local governments and their respective heads with regard to quarantine measures. For example, during the COVID-19 outbreak in Daegu in early 2020, Daegu’s health care and quarantine systems were quickly overwhelmed, and media coverage of the crisis was extensive. “Kwon Young-jin” (135 in 2020), the mayor of Daegu Metropolitan City, appeared in the news, as well as “Gwangju Metropolitan Government” (26 in 2020), which helped Daegu during its time of need. In July 2020, Daegu extended its helping hand to Gwangju when the latter faced a shortage of hospital beds due to the surge in COVID-19 cases. This act of solidarity and collaboration exemplifies the importance of interregional cooperation during public health emergencies.

Furthermore, the enactment of local government ordinances aimed at supporting essential workers, including nurses, in a non–face-to-face environment, as necessitated by the COVID-19 pandemic, is noteworthy. In this context, “Chong Won-o” (76 in total), head of the Seongdong-gu District Office in Seoul, enacted ordinances on safety measures, web-based work-support systems, and psychological counseling for essential workers such as nurses.

#### Economic and Political Section

The main sources of the economic news were related to free trade agreements (FTA). Unions for youth and women employment and corporate sources were also prominently featured. President “Park Geun-hye” (24 in total), who emphasized economic development by Korean nurses dispatched to Germany in the 1960s and 1970s, played a pivotal role as the primary source of economic development. During the COVID-19 pandemic, local governments became important in the economic sector.

Regarding the political section, the main sources have traditionally been the president and politicians affiliated with major political parties (ie, Ha Tae-keung, 79 in total). In the context of the COVID-19 pandemic, these sources emerged as a major source, as President “Moon Jae-in” (577 in total) engaged in a verbal dispute over a post on Facebook that praised the diligent efforts of nurses.

### Semantic Network Analysis: News Source-Topic Network Analysis

**Figure 2:**
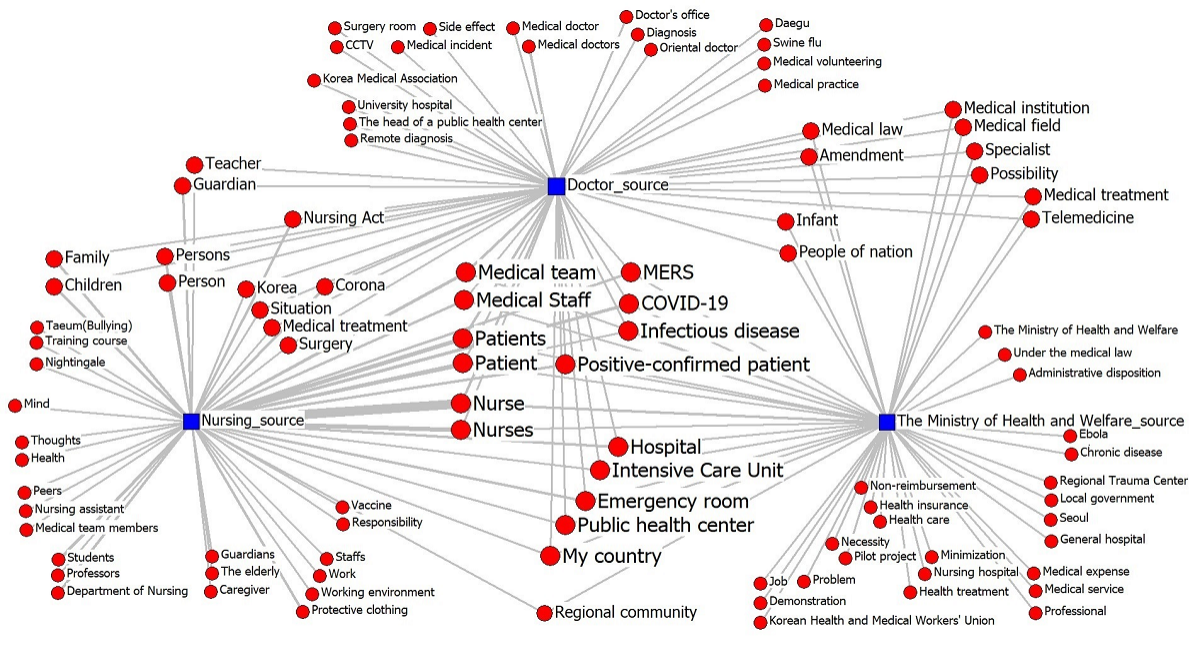
Topic network analysis by news sources. CCTV: closed-circuit television; MERS: Middle East respiratory syndrome.

A visualization of the news source-topic network for the top 50 topic words by health care source is shown in Figure 2. The analysis revealed 15 common topic words that were prominent among nurses, doctors, and the MOHW. The top common terms encompassed general health-related topics such as “nurses,” “patients,” “medical staff,” and “hospitals.” Notably, pandemic-related topics like “COVID-19,” “MERS” “confirmed cases,” and “infectious diseases” also featured prominently as shared interests.

“Nursing Bill” emerged as an issue of high interest among sources from the nursing and doctor groups although the topic did not garner significant attention from MOHW sources. Interestingly, both doctors and MOHW sources exhibited common interests in “the Medical Act” and “amendments,” although nurses mentioned the Medical Act less frequently. Moreover, the nurse group and MOHW sources shared a major concern with regard to the “community,” indicating a consensus on the importance of expanding public health care in the community.

Nurses demonstrated a distinct interest in education-related themes, such as “students” and “nursing department,” as well as concerns regarding poor working conditions, including “protective clothing” and “working environment.” Additionally, professional pride was evident through terms like “sense of mission” and “nightingale.” Unfortunately, workplace harassment, represented by the term “burning,” has emerged as an important issue for nurses.

Conversely, physician sources displayed a particular interest in “operating room” and “closed-circuit television (CCTV).” Doctors voiced opposition to the implementation of closed-circuit television surveillance in operating rooms to prevent medical errors. Furthermore, they expressed concerns about “telemedicine” and “Oriental medicine,” fearing potential infringement of doctors’ status. Their apprehension lies in the possibility that nondoctor medical practitioners, such as nurses and herbalists, may perform medical treatments and thereby exclude doctors through telemedicine platforms.

### Quote Analysis

#### Social Sections

The timeline and main topics of interest in articles related to nurses are detailed in Multimedia Appendix 6. Nurses have been portrayed in many ways in the social sector, and these portrayals have encompassed various roles that range from heroes to workers to survivors of abuse and even to perpetrators.

Nurses have been portrayed as heroes and dedicated nightingales who have been fighting at the frontlines of epidemic prevention to stop the spread of various infectious diseases—from swine flu and MERS to COVID-19. Such portrayals frequently emphasize courage, compassion, and dedication [1]. Besides their role in epidemic prevention, nurses’ heroic images extend beyond this domain and encompass their initial responses to various mass casualty incidents. An illustrative example is the Itaewon tragedy of 2022, wherein the remarkable efforts of nurses were at the forefront. One nurse said, “I think I performed CPR so extensively that I lost count of the number of individuals I attended to” [21].

Beyond this heroic image, other reports have emerged which indicate that nurses face unfavorable working conditions like those of laborers. These conditions are characterized by excessive workloads, inadequate compensation, and lack of recognition of their invaluable contributions. Nurses’ voices in the social media coverage extend beyond merely conveying the difficulties associated with nursing work. Furthermore, nurses serve as valuable sources who articulate calls for policy alternatives or provide evaluations of existing policies. This multifaceted portrayal of nurses, which highlights their roles as workers, experts, and heroes, is observed both in the national and international media [22,23].

Furthermore, nurses appeared in the media as suspects or perpetrators of medical accidents, such as the death of a newborn at Ewha Womans University Mokdong Hospital in 2017, and as key sources in cases involving surrogate doctors. However, nurses are not responsible for medical errors and are described as enforcers who function under the supervision of hospitals and doctors.

Nurses have progressed beyond communicating difficulties in the nursing workplace and whistleblowing incidents to initiating calls for policy alternatives and evaluating ongoing policies in media reports. In particular, the Korean Nursing Association emerged as a major source, besides nurses, when discussions of the Nursing Care Act began to be earnestly covered in the media. Simultaneously, the Korean Medical Association strongly opposed the nursing law.

#### Local Sections

In the local section, there was further emphasis on poor health care and working conditions, which were revealed in the social section. Primary sources associated with “hospitals” have voiced concern with regard to the challenges that are encountered within local health care facilities, particularly in socioeconomically disadvantaged regions. Throughout different infectious disease outbreaks, including the pandemic, MERS, and COVID-19, the focal points of the discussion included the burdensome workloads imposed on nurses, inadequate staffing levels, and insufficient resources, including facilities and equipment.

This situation underscores the need for an expanded role of nurses within the community, greater recognition of the nursing profession, and enhanced public awareness of health care issues. Public and governmental sources, such as the MOHW, nursing organizations, and unions, have advocated the need for increased investment in public health care. The provision of health care services based solely on market principles is limited to regions characterized by smaller populations and relatively lower incomes. Consequently, the government has endeavored to fortify local public health care by emphasizing national university hospitals and public health centers as central hubs.

Various measures have been pursued to achieve the above-described goal. These measures include legislative initiatives for the establishment of community and professional nurse-systems, as well as the expansion and reinforcement of the nursing profession’s role through mechanisms such as the nursing grade system and the expansion of nursing schools. Moreover, public support has been provided to bolster these efforts. Although it is acknowledged that challenges, including trial and error, disagreements within the nursing profession, and conflicts with other health care organizations, such as the Korean Medical Association, have emerged during this process, the media generally presents the strengthening of local public health care as being a step toward aligning with the enhanced status of the nursing profession.

In the case of the closure of Jinju Public Medical Center in 2013 and the health care union’s call for a general strike in 2021, “unions” appear as the main source of information, calling for the strengthening of public health care. The nursing profession, along with various sources from government departments, such as the MOHW, emphasizes the need to bolster public health care in local media discourse. Media reports portray the government as actively seeking to fortify local public health care with a focus on local university hospitals and public health centers. Specifically, various policies were mentioned, such as the introduction of nurse practitioners, visiting nurses, dispatch nurses, nurses in training, part-time nurses, comprehensive nursing services, a nursing grading system, more nursing schools, and public health nurses. These policies aim to improve the local health care environment by expanding and strengthening the roles of nurses and providing public support.

#### Economy Sections

Between 2005 and 2007, the opening of the health care market emerged as a significant economic agenda within the context of the Korea-US FTA. Consequently, notable figures such as Kim Jong-hoon, who was the Korea’s chief representative in the US-Korea FTA; Kim Hyun-Jong, who served as the head of the Trade Negotiations Division at the Ministry of Foreign Affairs; and Wendy Cutler, who was the US chief representative, became a key source in the economy section of the media.

Unions represent another significant source in the economic sector, particularly in relation to the interests of nurses. Various unions, including major hospital unions, the National Healthcare Workers’ Union, the Democratic Trade Union Confederation (to which the National Healthcare Workers’ Union is affiliated), and the Public Transport Workers’ Union Medical Solidarity Headquarters (affiliated to the Public Transport Workers’ Union) have been actively engaged in shaping the discourse on nurses’ issues within the broader framework of labor concerns. These unions shed light on key topics, such as insufficient nurse recruitment, the growing trend of casualization through the introduction of part-time work, and substandard working conditions for women employees. The sources from the “union” sector on the economy section of the media provide valuable insights that reflect a broader sense of solidarity with the collective of nurses.

In addition to the heroic portrayal of nurses during the pandemic, the use of nurse imagery in economic development is observed, as exemplified by the depiction of the “Korean nurses in West Germany” as an economic development hero. President Park Geun-hye has associated the concepts of “Korean nurses to West Germany,” along with “Korean miners to West Germany” and “Middle Eastern construction workers,” with overseas employment initiatives that are aimed at combatting youth unemployment and interlinking nurses within the economic policy direction.

#### Political Sections

Politicians from major parties and heads of government ministries, including Moon Jae-in, have emerged as key sources. During the COVID-19 pandemic in 2020 and 2021, President Moon found himself amid a controversy. The president is expected to express gratitude to the health care workers, particularly nurses, doctors, and pharmacists.

His emphasis on the nurses’ hard work became a point of political contention. A notable example was the 2020 controversy surrounding the division of medical staff at a time when doctors were on strike to protest the government’s plans to increase the number of seats in public medical schools and establish new public medical schools. On September 2, 2020, President Moon wrote a Facebook post stating, “Nurses are silently guarding medical sites where doctors, including specialists, have left,” and highlighting that the majority of medical staff referred to as “doctors” were actually nurses. These statements drew criticism from members of the opposite People’s Power Party, such as the representative Ha Tae-kyung and spokesperson Kim Eun-hye, who accused President Moon of dividing the medical staff based on their profession. In contrast, the ruling Democratic Party of Korea and representative Ko Goo-jung refuted the division of the medical staff frame and countered that it was the People’s Power Party that created a division between the government and medical staff. Furthermore, debates surrounding the authorship of Facebook posts added another layer of controversy to the situation.

In the political section, the news articles featured a range of stakeholders, including the nursing community, who voiced their perspectives on the Nursing Bill. However, rather than providing an in-depth analysis of the policy’s specific content, media coverage has primarily emphasized the conflict between nurses and doctors with regard to the enactment of the bill, as well as the debates between President Moon Jae-in and the opposition parties.

The increased media attention on the nursing law enactment began in earnest under President Moon Jae-in’s administration after the COVID-19 pandemic, and the controversy surrounding the enactment of the nursing law that represented the ongoing conflict between the Korean Nurses Association and the Korean Medical Association has also received significant attention. The conflict over the Nursing Bill was manifested in various ways, such as the resolution of a general strike by nurses in 2021 and the boycott of the national nursing examination in 2022. The strategies used by nurses in their struggle, which encompassed labor disputes with unions and profession-wide refusal to participate in the national examination, reflect the dual nature of their roles as both workers and professionals.

Following the public’s widespread recognition and support for the dedicated efforts of nurses during the COVID-19 pandemic, the Nursing Bill has resurfaced as an important issue that has led both the major presidential candidates in the 2022 election to pledge their commitment toward enacting nursing legislation. However, a divergence in approach has become apparent, with the newly elected People’s Power Party expressing caution following internal disagreements. Contrarily, the Democratic Party affirmed its intent to pursue the enactment of the nursing legislation. This eventually resulted in the president vetoing the legislation that had been passed by the opposition-led National Assembly in 2023.

## Discussion

### Principal Findings

This study focused on analyzing media coverage of the nursing agenda in South Korea over an 18-year period starting in 2005 and examined the coverage patterns, sources responsible for reporting, and quotes in articles, both annually and across different sections. To achieve this objective, a large data set comprising nurse-related articles from BigKinds was used.

In sum, 128,339 articles from 54 media outlets on nursing-related issues were analyzed. The news was mainly covered in social and local sections. According to news source network analysis, 445 main sources who had made the top 20 list at least once by year and section were selected. Multimedia Appendices 2-5 show the top 20 sources by year and section.

The news source-topic network analysis highlights nursing legislation as a common concern for intense conflict between doctors and nurses. The “Nursing Bill” emerged as a common interest among nurses and doctors although the topic did not garner considerable attention from the MOHW.

This study aimed to examine the drivers of the social, local, economic, and political agendas emphasized in the media by the analysis of main sources and their quotes. In the social section, various issues were covered, including the COVID-19 response, workplace bullying, and nursing bills. On the local level, poor working conditions and strengthening public health care were key issues. Considering the economic aspect, labor issues and overseas work were hot topics. On the political aspect, conflicts between nurses and doctors over the nursing bills and the resulting controversy were crucial. This study revealed the media’s evolving portrayal of nurses over time. Nurses are portrayed in various roles, such as heroes, workers, survivors of abuse, and even perpetrators, across different topics covered by the media. Nonetheless, in the economic and political spheres, nurses’ voices tend to diminish. Media coverage of the enactment of nursing laws tends to move on confrontations between political parties.

### Comparison to Prior Work

Aber and Hawkins [24] undertook a comprehensive examination of advertisements featured in medical and nursing journals. Their findings elucidated a prevailing depiction of nurses in print media, where they were predominantly portrayed as ornamental figures, sexualized objects, or ancillaries to doctors. Hoyle et al [25] embarked on a comprehensive exploration of nurses’ perceptions regarding the media’s influence on the public understanding of the nursing profession. They discerned recurrent themes, notably the media’s negative portrayal of the nursing profession—a sentiment echoed in other studies [26]. In contrast, the severe acute respiratory syndrome crisis served as a crucial juncture in the media representation of nurses, akin to the role of the COVID-19 pandemic in South Korea [9,10,27]. These preceding studies have demonstrated the media’s paramount influence in shaping societal perceptions of nursing.

Our study underscores a more dynamic evolution of media portrayals pertaining to nurses. During health crises, the media consistently lionized nurses, portraying them as the unsung heroes on the frontlines. However, media portrayals of nurses have undergone fundamental transformations, mirroring the societal evolutions and the multifaceted challenges encountered by the nursing fraternity. With South Korea confronting the challenges of an aging demographic, there is an evident amplification in nurses’ community-based responsibilities. This evolution signifies a transition from a traditionally passive representation to a more proactive and central role transcending their conventional subordinate position relative to doctors.

Media coverage can influence not only the image of nurses but also their working conditions, the structure of the health care workforce, and the legislative process [8,26,28]. The paradigm shift in the media agenda has precipitated heightened political and juridical tensions between the medical and nursing professions. Conflicts between health care professionals, as evidenced by the political dimension analysis, can impede policymaking and result in policy confusion due to politicization. Previous instances of policy confusion within the health care community, including health insurance integration, medical division of labor, insurance finance policy, and reimbursement policy, have been attributed to conflicting interests and backlash from targeted groups [29,30]. Moreover, these factors have been reflected in the media representations.

### Limitations

This study provides a comprehensive analysis of media coverage of nurses over an 18-year period since 2005, with a focus on the sources. However, this study has several limitations.

First, the study analyzed only major newspapers and broadcast media that are listed on BigKinds, which means that data from health care journals that were not included in BigKinds could not be analyzed. Future research could compare agenda-related differences between the mass media and health care professional journals to explore how variations in general and public agendas influence policy agendas. Additionally, it would be valuable to examine discussions on platforms, such as Twitter and blogs, to capture a broader range of perspectives.

Second, as this study’s analysis was based on all articles containing the keyword “nurses,” this strategy limited the ability to descriptively delve into specific agendas. A follow-up study could focus on analyzing a specific agenda, such as the Nursing Bill, over an extended timeframe to gain a more nuanced understanding of policymaking through media channels.

Third, this study implies that health care policy is not solely based on scientific advancements or socioeconomic justifications but is inherently political. Strategic media outreach and policy research are necessary to elucidate policy directions that nursing communities aim to pursue.

### Conclusions

This study used a combination of NLP and semantic network analyses to examine how nurse-related issues were covered by the media, specifically focusing on sources and quotes. The media plays a crucial role in highlighting various aspects of nursing practice and nurses, thereby contributing to political engagement and policy activism. However, policy formulation processes to solve nursing issues have not been adequately reported in South Korea.

When engaging with the media to promote nursing policy, it is crucial to navigate the politicization of issues and focus on how policy matters are prioritized on the agenda. As advocates for public health, nurses are responsible for actively engaging in legislative and policymaking processes from a political standpoint [31-33]. However, nurses may not fully realize the extent to which nursing practice relies on public policy decisions and their potential to shape those decisions [34,35]. Nurses should participate in various activities at different levels, such as attending public forums and giving media interviews, to voice their professional views and positions as advocates [36,37]. Nurses need political competence to address the broader determinants of health, effectively intervene in culturally diverse societies, collaborate in developing humane health care systems, and bring nursing values to policy discussions [38].

## Appendix

Multimedia Appendix 1 The size, number of edges, and density of 72 news source networks.

Multimedia Appendix 2 Top 20 news sources by year in the social section.

Multimedia Appendix 3 Top 20 news sources by year in the local section.

Multimedia Appendix 4 Top 20 news sources by year in the economy section.

Multimedia Appendix 5 Top 20 news sources by year in the political section.

Multimedia Appendix 6 The timeline and main agenda in nurse articles.

## Abbreviations

BERT: bidirectional encoder representations from transformers
FTA: free trade agreements
IRB: institutional review board
MERS: Middle East respiratory syndrome
MOHW: Ministry of Health and Welfare
NER: named entity recognition
NLP: natural language processing
RQ: research question

## Data Availability

Data are not available to share due to Department of Veterans Affairs and ethical retractions; however, annotation instructions and vocabulary are available upon request..
