# Supplementary material for: Agendas on Nursing in South Korea Media: Natural Language Processing and Network Analysis of News From 2005 to 2022
Source: J Med Internet Res. 2024 Mar 19;26:e50518. doi: 10.2196/50518 (PMC10988384; doi:10.2196/50518)
Supplement: Multimedia Appendix 1 [file jmir_v26i1e50518_app1.docx]

Appendix 1. The size, number of edges, and density of 72 news source networks.

| Year | Size | | | | Edge | | | | Density | | | |
| --- | --- | --- | --- | --- | --- | --- | --- | --- | --- | --- | --- | --- |
|  | Social affairs | Local | Economy | Politics | Social affairs | Local | Economy | Politics | Social affairs | Local | Economy | Politics |
|  |  |  |  |  |  |  |  |  |  |  |  |  |
| 2005 | 1242 | 485 | 170 | 98 | 2236 | 777 | 256 | 139 | 0.00290 | 0.00662 | 0.01782 | 0.02924 |
| 2006 | 1279 | 425 | 264 | 109 | 2193 | 643 | 412 | 152 | 0.00268 | 0.00714 | 0.01187 | 0.02582 |
| 2007 | 1484 | 544 | 247 | 203 | 2625 | 878 | 373 | 284 | 0.00239 | 0.00594 | 0.01228 | 0.01385 |
| 2008 | 1791 | 739 | 249 | 131 | 3629 | 1300 | 368 | 213 | 0.00226 | 0.00477 | 0.01192 | 0.02501 |
| 2009 | 2357 | 898 | 347 | 219 | 5309 | 1863 | 566 | 367 | 0.00191 | 0.00463 | 0.00943 | 0.01537 |
| 2010 | 2203 | 846 | 228 | 216 | 4819 | 1553 | 317 | 337 | 0.00199 | 0.00434 | 0.01225 | 0.01451 |
| 2011 | 2600 | 971 | 548 | 331 | 5389 | 1832 | 851 | 572 | 0.00159 | 0.00389 | 0.00568 | 0.01047 |
| 2012 | 2604 | 955 | 378 | 422 | 5582 | 1748 | 548 | 689 | 0.00165 | 0.00384 | 0.00769 | 0.00776 |
| 2013 | 3009 | 1099 | 490 | 264 | 6698 | 2125 | 766 | 450 | 0.00148 | 0.00352 | 0.00639 | 0.01296 |
| 2014 | 3357 | 1156 | 350 | 418 | 8587 | 2353 | 563 | 789 | 0.00152 | 0.00352 | 0.00922 | 0.00905 |
| 2015 | 4344 | 1639 | 374 | 421 | 12,373 | 3146 | 586 | 750 | 0.00131 | 0.00234 | 0.00840 | 0.00848 |
| 2016 | 3441 | 1337 | 401 | 591 | 7006 | 2279 | 585 | 962 | 0.00118 | 0.00255 | 0.00729 | 0.00552 |
| 2017 | 3632 | 1678 | 526 | 598 | 7868 | 2870 | 736 | 1,303 | 0.00119 | 0.00204 | 0.00533 | 0.00730 |
| 2018 | 4354 | 1910 | 482 | 372 | 10,150 | 3855 | 698 | 559 | 0.00107 | 0.00211 | 0.00602 | 0.00810 |
| 2019 | 4194 | 1707 | 400 | 318 | 8944 | 3264 | 567 | 462 | 0.00102 | 0.00224 | 0.00711 | 0.00917 |
| 2020 | 7001 | 3692 | 627 | 1,100 | 20,461 | 9997 | 955 | 3,299 | 0.00084 | 0.00147 | 0.00487 | 0.00546 |
| 2021 | 6093 | 2600 | 523 | 870 | 16,705 | 5656 | 756 | 2,023 | 0.00090 | 0.00167 | 0.00554 | 0.00535 |
| 2022 | 4162 | 1846 | 423 | 843 | 9633 | 3478 | 576 | 1,570 | 0.00111 | 0.00204 | 0.00645 | 0.00442 |
