# Supplementary material for: Agendas on Nursing in South Korea Media: Natural Language Processing and Network Analysis of News From 2005 to 2022
Source: J Med Internet Res. 2024 Mar 19;26:e50518. doi: 10.2196/50518 (PMC10988384; doi:10.2196/50518)
Supplement: Multimedia Appendix 2 [file jmir_v26i1e50518_app2.docx]

Appendix 2. Top 20 News Sources by Year in the Social Section.

| Source | Year | | | | | | | | | | | | | | | | | | Grand  Total |
| --- | --- | --- | --- | --- | --- | --- | --- | --- | --- | --- | --- | --- | --- | --- | --- | --- | --- | --- | --- |
|  | 2005 | 2006 | 2007 | 2008 | 2009 | 2010 | 2011 | 2012 | 2013 | 2014 | 2015 | 2016 | 2017 | 2018 | 2019 | 2020 | 2021 | 2022 |  |
|  | Degree centrality^a^ | | | | | | | | | | | | | | | | | |  |
| Nurse | 28 | 37 | 55 | 51 | 65 | 43 | 60 | 41 | 74 | 80 | 117 | 130 | 136 | 176 | 124 | 351 | 332 | 191 | 2091 |
| Hospital | 25 | 38 | 40 | 34 | 108 | 51 | 56 | 45 | 54 | 75 | 172 | 110 | 117 | 149 | 100 | 258 | 222 | 136 | 1790 |
| Labor union | 5 | 6 | 20 | 7 | 7 | 12 | 29 | 5 | 89 | 48 | 66 | 23 | 45 | 112 | 66 | 106 | 229 | 96 | 971 |
| Police | 9 | 14 | 6 | 13 | 27 | 15 | 9 | 55 | 22 | 72 | 33 | 47 | 95 | 158 | 97 | 66 | 77 | 54 | 869 |
| Ministry of Health and Welfare | 9 | 11 | 39 | 19 | 10 | 11 | 27 | 36 | 60 | 59 | 63 | 77 | 43 | 91 | 40 | 49 | 48 | 48 | 740 |
| Doctor | 6 | 20 | 16 | 12 | 41 | 23 | 29 | 20 | 20 | 29 | 46 | 39 | 17 | 95 | 49 | 82 | 105 | 37 | 686 |
| Government |  |  | 11 | 23 | 27 | 6 | 20 |  | 3 | 15 | 28 | 20 | 15 | 25 | 27 | 182 | 144 | 74 | 620 |
| Department of Justice | 9 | 6 | 11 | 18 | 13 | 12 | 15 | 36 | 24 | 46 | 19 | 54 | 28 | 33 | 99 | 65 | 69 | 41 | 598 |
| Central Disaster Management Headquarters |  |  |  |  |  |  |  |  |  |  |  |  |  |  |  | 199 | 242 | 100 | 541 |
| Medical team | 12 | 8 | 5 | 16 | 20 | 12 | 16 | 8 | 6 | 26 | 45 | 8 | 11 | 34 | 14 | 89 | 104 | 22 | 456 |
| Moon Jae-in, the former President of Korea |  |  |  |  |  |  |  |  |  |  |  |  | 18 |  | 19 | 121 | 175 | 12 | 345 |
| Korean Medical Association |  |  | 7 | 14 |  |  | 5 | 6 | 7 | 13 | 7 |  | 13 | 46 | 34 | 115 | 34 | 42 | 343 |
| Medical field |  |  | 3 |  |  |  | 3 | 6 | 11 | 11 | 36 | 31 | 23 | 60 | 30 | 37 | 29 | 37 | 317 |
| Local resident | 8 | 12 | 4 | 11 | 6 |  | 9 | 8 | 18 | 18 | 41 | 18 | 15 | 16 | 9 | 71 | 17 | 27 | 308 |
| Public health center | 7 |  | 9 | 14 | 21 | 11 | 8 | 10 | 10 | 14 | 32 | 11 | 11 | 25 | 8 | 30 | 41 | 11 | 273 |
| Prosecution |  | 5 | 5 |  | 6 |  | 13 | 26 | 22 | 13 | 6 | 16 | 15 | 9 | 21 | 28 | 44 | 7 | 236 |
| Korean Nursing Association |  |  |  |  | 4 |  | 5 | 11 |  | 20 | 4 |  | 4 | 22 | 17 | 69 | 23 | 54 | 233 |
| Seoul Metropolitan Government |  |  |  |  |  |  |  | 5 | 15 | 15 | 12 |  | 9 | 8 | 28 | 89 | 41 | 8 | 230 |
| Chung Sye-kyun, the Prime Minister |  |  |  |  |  |  |  |  |  |  |  |  |  |  |  | 116 | 109 |  | 225 |
| Supreme court |  |  | 7 | 9 |  | 3 | 4 | 9 | 8 | 8 | 9 | 5 | 13 | 27 | 21 | 27 | 34 | 37 | 221 |
| a. The gradation in green indicates the degree of importance of the source, with the greener being the more important. | | | | | | | | | | | | | | | | | | | |
