# Supplementary material for: Agendas on Nursing in South Korea Media: Natural Language Processing and Network Analysis of News From 2005 to 2022
Source: J Med Internet Res. 2024 Mar 19;26:e50518. doi: 10.2196/50518 (PMC10988384; doi:10.2196/50518)
Supplement: Multimedia Appendix 3 [file jmir_v26i1e50518_app3.docx]

Appendix 3. Top 20 News Sources by Year in the Local Section.

| Source | Year | | | | | | | | | | | | | | | | | | Grand  Total |
| --- | --- | --- | --- | --- | --- | --- | --- | --- | --- | --- | --- | --- | --- | --- | --- | --- | --- | --- | --- |
|  | 2005 | 2006 | 2007 | 2008 | 2009 | 2010 | 2011 | 2012 | 2013 | 2014 | 2015 | 2016 | 2017 | 2018 | 2019 | 2020 | 2021 | 2022 |  |
|  | Degree centrality^a^ | | | | | | | | | | | | | | | | | |  |
| Hospital | 12 | 10 | 8 | 12 | 42 | 9 | 24 | 9 | 19 | 34 | 38 | 20 | 54 | 36 | 39 | 103 | 70 | 38 | 577 |
| Nurse | 3 | 9 | 8 |  | 15 | 9 | 7 | 12 | 9 | 9 | 13 | 21 | 35 | 29 | 17 | 107 | 78 | 25 | 406 |
| Local resident | 8 | 9 | 3 | 5 | 3 | 8 | 10 | 9 | 12 | 13 | 36 | 15 | 23 | 21 | 16 | 51 | 35 | 12 | 289 |
| Police | 7 |  | 3 | 3 | 8 |  | 6 |  | 10 | 17 | 9 | 22 | 13 | 58 | 34 | 41 | 18 | 32 | 281 |
| Labor union | 5 | 3 |  |  | 4 |  | 4 |  | 37 | 10 | 11 | 10 | 8 | 19 | 15 | 19 | 36 | 26 | 207 |
| Central Disaster Management Headquarters |  |  |  |  |  |  |  |  |  |  |  |  |  |  |  | 71 | 49 | 30 | 150 |
| Kwon Young-jin, Mayor of Daegu |  |  |  |  |  |  |  |  |  |  |  |  |  |  |  | 135 | 3 |  | 138 |
| Government |  |  |  |  |  |  |  |  |  | 3 | 7 |  |  | 9 | 3 | 83 | 18 | 15 | 138 |
| Ministry of Health and Welfare |  |  | 3 |  |  |  | 4 | 3 | 11 | 15 | 18 | 7 | 7 | 14 | 11 | 21 | 12 | 8 | 134 |
| Public health center | 5 |  | 12 | 4 | 10 | 7 | 6 |  |  | 4 | 11 | 5 | 9 | 14 | 8 | 15 | 19 | 4 | 133 |
| Daegu Metropolitan Government |  |  |  | 6 |  |  |  |  |  | 12 | 3 |  |  | 6 | 5 | 83 | 13 | 3 | 131 |
| Medical team |  |  | 3 | 4 | 7 |  |  | 3 |  |  | 16 |  |  |  | 3 | 29 | 17 | 14 | 96 |
| General coordinator |  |  |  |  |  |  |  |  |  |  |  |  |  |  |  | 88 | 8 |  | 96 |
| Medical field |  |  |  |  |  |  |  |  | 9 |  | 7 | 4 | 5 | 10 | 14 | 23 | 13 | 6 | 91 |
| Gyeonggi-do |  | 10 |  |  | 3 | 4 |  |  |  |  |  |  | 5 | 10 | 4 | 46 | 3 |  | 85 |
| Chong Won-o, Mayor of Seongdong-gu |  |  |  |  |  |  |  |  |  |  |  |  |  | 3 | 5 | 7 | 39 | 22 | 76 |
| Nursing hospital |  |  |  |  |  |  |  | 4 | 5 | 9 | 3 |  |  | 6 |  | 6 | 23 | 15 | 71 |
| Korean Medical Association |  |  |  |  |  |  |  |  |  |  |  |  |  | 15 |  | 44 | 6 | 3 | 68 |
| Gwangju Metropolitan Government |  |  |  |  |  |  |  | 3 | 4 | 3 | 4 | 4 | 6 |  |  | 26 | 13 | 3 | 66 |
| Kim Seon-gap, Mayor of Gwangjin-gu |  |  |  |  |  |  |  |  |  |  |  |  |  |  |  |  | 33 | 26 | 59 |
| a. The gradation in green indicates the degree of importance of the source, with the greener being the more important. | | | | | | | | | | | | | | | | | | | |
