# Supplementary material for: Agendas on Nursing in South Korea Media: Natural Language Processing and Network Analysis of News From 2005 to 2022
Source: J Med Internet Res. 2024 Mar 19;26:e50518. doi: 10.2196/50518 (PMC10988384; doi:10.2196/50518)
Supplement: Multimedia Appendix 4 [file jmir_v26i1e50518_app4.docx]

Appendix 4. Top 20 News Sources by Year in the Economy Section.

| Source | Year | | | | | | | | | | | | | | | | | | Grand  Total |
| --- | --- | --- | --- | --- | --- | --- | --- | --- | --- | --- | --- | --- | --- | --- | --- | --- | --- | --- | --- |
|  | 2005 | 2006 | 2007 | 2008 | 2009 | 2010 | 2011 | 2012 | 2013 | 2014 | 2015 | 2016 | 2017 | 2018 | 2019 | 2020 | 2021 | 2022 |  |
|  | Degree centrality^a^ | | | | | | | | | | | | | | | | | |  |
| Nurse | 5 | 5 | 8 | 4 | 8 | 8 | 4 | 4 | 4 |  | 10 | 5 | 4 | 5 |  | 8 | 8 | 12 | 102 |
| Government |  | 10 | 9 |  | 6 |  | 18 |  | 9 | 7 | 4 |  | 5 | 11 |  |  | 3 | 8 | 90 |
| Police |  |  |  |  |  |  |  | 10 |  | 7 | 9 | 6 |  |  |  |  |  |  | 32 |
| Korean Confederation of Trade Unions |  |  |  |  | 12 |  |  |  |  | 4 | 5 |  | 5 |  |  |  |  |  | 26 |
| Ministry of Employment and Labor | 7 |  |  |  | 15 |  |  |  |  |  |  |  |  |  |  |  |  | 4 | 26 |
| Samsung Electronics |  |  |  |  |  | 4 | 7 |  |  |  |  |  |  |  |  | 11 | 3 |  | 25 |
| Seoul Metropolitan Government |  |  |  |  |  |  |  |  |  |  |  |  |  |  | 6 |  | 10 | 8 | 24 |
| Park Geun-hye (the former president of Korea) |  |  |  |  |  |  |  |  | 9 | 7 | 5 | 3 |  |  |  |  |  |  | 24 |
| Wall Street Journal |  |  |  |  |  |  |  |  |  |  |  |  | 12 |  |  | 8 | 4 |  | 24 |
| Business field |  |  |  |  |  |  |  |  | 13 |  |  |  |  |  |  | 5 | 5 |  | 23 |
| Professor Lee Byoung-Hoon, Chunag-Ang University |  |  |  |  |  |  | 10 |  |  |  | 7 |  |  |  |  |  | 5 |  | 22 |
| Ministry of Health and Welfare |  |  | 4 |  |  |  | 5 |  | 3 | 3 |  |  |  | 7 |  |  |  |  | 22 |
| International Monetary Fund |  |  |  |  |  |  | 9 |  |  | 3 |  |  |  |  |  | 10 |  |  | 22 |
| Insurance industry |  |  |  |  |  |  |  | 11 |  |  |  |  | 3 |  | 4 | 3 |  |  | 21 |
| Kim Jong-hoon, Minister for Trade |  | 18 | 3 |  |  |  |  |  |  |  |  |  |  |  |  |  |  |  | 21 |
| CJ Group |  |  |  |  |  |  |  | 6 |  |  | 3 |  | 12 |  |  |  |  |  | 21 |
| Human Resources Development Service of Korea |  | 6 | 8 |  |  |  |  |  |  |  |  |  |  | 6 |  |  |  |  | 20 |
| The Federation of Korean Industries |  |  |  |  |  |  | 3 |  |  |  | 5 |  | 6 |  |  | 6 |  |  | 20 |
| Ryu Gyeong-gi Mayor of Jungnang-gu |  |  |  |  |  |  |  |  |  |  |  |  |  |  |  |  | 8 | 12 | 20 |
| Kim Hyun-chong, Minister for Trade |  | 8 | 12 |  |  |  |  |  |  |  |  |  |  |  |  |  |  |  | 20 |
| a. The gradation in green indicates the degree of importance of the source, with the greener being the more important. | | | | | | | | | | | | | | | | | | | |
