# Supplementary material for: Agendas on Nursing in South Korea Media: Natural Language Processing and Network Analysis of News From 2005 to 2022
Source: J Med Internet Res. 2024 Mar 19;26:e50518. doi: 10.2196/50518 (PMC10988384; doi:10.2196/50518)
Supplement: Multimedia Appendix 5 [file jmir_v26i1e50518_app5.docx]

Appendix 5. Top 20 News Sources by Year in the Political Section.

| Source | Year | | | | | | | | | | | | | | | | | | Grand  Total |
| --- | --- | --- | --- | --- | --- | --- | --- | --- | --- | --- | --- | --- | --- | --- | --- | --- | --- | --- | --- |
|  | 2005 | 2006 | 2007 | 2008 | 2009 | 2010 | 2011 | 2012 | 2013 | 2014 | 2015 | 2016 | 2017 | 2018 | 2019 | 2020 | 2021 | 2022 |  |
|  | Degree centrality^a^ | | | | | | | | | | | | | | | | | |  |
| Moon Jae-in,  Former President of Korea |  |  |  |  |  |  |  |  |  |  |  |  | 66 | 13 | 17 | 288 | 151 | 42 | 577 |
| The Blue House |  |  |  |  | 4 | 7 |  |  | 6 | 3 | 12 | 35 | 22 | 3 | 7 | 86 | 73 | 8 | 266 |
| Nurse |  |  | 5 | 6 | 7 | 7 | 7 | 6 | 4 |  | 5 | 27 | 30 | 7 | 13 | 35 | 25 | 28 | 212 |
| Government |  |  | 11 | 4 | 11 | 7 | 6 | 30 | 6 | 11 | 4 |  | 14 | 3 | 3 | 45 | 24 | 7 | 186 |
| People’s Power Party |  |  |  |  |  |  |  |  |  |  |  |  |  |  |  | 45 | 57 | 46 | 148 |
| Democratic Party |  |  |  |  |  | 6 | 4 | 5 | 15 | 10 |  |  | 5 |  | 3 | 45 | 34 | 17 | 144 |
| Park Geun-hye, President of Korea |  |  |  |  |  |  |  |  | 24 | 29 | 22 | 39 |  |  |  |  |  |  | 114 |
| Ha Tae-keung, (People’s Power Party) |  |  |  |  |  |  |  |  |  |  |  |  |  |  |  | 79 |  |  | 79 |
| Kim Eun-hye, a spokesperson for the People’s Power Party |  |  |  |  |  |  |  |  |  |  |  |  |  |  |  | 64 | 9 |  | 73 |
| Korean Nurses Association |  |  |  |  |  |  |  | 5 |  |  |  |  | 3 |  | 5 | 22 | 8 | 27 | 70 |
| Korean Medical Association |  |  |  | 3 |  |  |  |  |  | 5 |  | 4 |  | 11 |  | 7 | 13 | 25 | 68 |
| Prime Minister Chung Se-kyun |  |  |  |  |  |  |  |  |  |  |  |  |  |  |  | 19 | 47 |  | 66 |
| Hong Joon-pyo, Member of the National Assembly | 14 |  | 3 |  |  |  |  |  |  |  |  |  |  |  |  | 38 | 8 |  | 63 |
| National Assembly |  |  |  |  |  |  |  |  |  | 7 | 16 | 4 |  |  |  | 11 | 22 |  | 60 |
| Yun Hee-suk (People’s Power Party) |  |  |  |  |  |  |  |  |  |  |  |  |  |  |  | 52 | 7 |  | 59 |
| National Assembly | 21 | 4 | 16 | 3 |  | 3 |  |  |  |  |  |  |  |  |  |  |  | 10 | 57 |
| Ahn Cheol-Soo, a leader of the People's Party |  |  |  |  |  |  |  |  |  |  |  |  |  |  |  | 54 | 3 |  | 57 |
| Ko Min-jung (Democratic Party) |  |  |  |  |  |  |  |  |  |  |  |  |  |  |  | 42 |  | 15 | 57 |
| Labor union |  |  |  |  |  |  |  |  | 3 |  |  | 8 | 4 | 5 |  |  | 28 | 6 | 54 |
| Ministry of Health and Welfare |  |  | 4 |  |  |  |  | 9 | 3 |  | 11 | 9 |  | 4 |  |  | 5 | 7 | 52 |
| a. The gradation in green indicates the degree of importance of the source, with the greener being the more important. | | | | | | | | | | | | | | | | | | | |
