# Supplementary material for: Agendas on Nursing in South Korea Media: Natural Language Processing and Network Analysis of News From 2005 to 2022
Source: J Med Internet Res. 2024 Mar 19;26:e50518. doi: 10.2196/50518 (PMC10988384; doi:10.2196/50518)
Supplement: Multimedia Appendix 6 [file jmir_v26i1e50518_app6.docx]

Appendix 6. The timeline and main agenda in nurse articles

| Year | Social & local section | Economy & politics section |
| --- | --- | --- |
|  |  |  |
| 2005 | - | FTA |
| 2006 | - | FTA |
| 2007 | - | FTA |
| 2008 | - | - |
| 2009 | H1N1 influenza, working condition in hospital | - |
| 2010 | - | - |
| 2011 | - | - |
| 2012 | - | - |
| 2013 | Closing Jinju Medical Center | - |
| 2014 | - | Korean Nurses Dispatched to Germany |
| 2015 | MERS, working condition in hospital | - |
| 2016 | Bullying, working condition in hospital | - |
| 2017 | Bullying, working condition in hospital, medical malpractice | - |
| 2018 | Suicide by bullying, medical malpractice | - |
| 2019 | Suicide by bullying, working condition in hospital | - |
| 2020 | COVID-19, collaboration between local hospitals | Strike by medical doctors,  dispute over a post on Facebook by President Moon |
| 2021 | COVID-19, Suicide by bullying, strike by healthcare union, supporting nurses | Conflicts between nurses and doctors on the nursing law |
| 2022 | Itaewon crush | Presidential election, pledge to enact nursing law |
